# circSLC4A7 accelerates stemness and progression of gastric cancer by interacting with HSP90 to activate NOTCH1 signaling pathway

**DOI:** 10.1038/s41419-023-05976-w

**Published:** 2023-07-20

**Authors:** Yang Hui, Yuan Wenguang, Shang Wei, Wang Haoran, Ning Shanglei, Liu Ju

**Affiliations:** 1 Center for post-doctoral studies, Shandong University of Traditional Chinese Medicine, Jinan, Shandong 250012 China; 2 Department of General Surgery, The First Affiliated Hospital of Shandong First Medical University & Shandong Provincial Qianfoshan Hospital, Key Laboratory of Metabolism and Gastrointestinal Tumor, the First Affiliated Hospital of Shandong First Medical University, Key Laboratory of Laparoscopic Technology, the First Affiliated Hospital of Shandong First Medical University, Shandong Medicine and Health Key Laboratory of General Surgery, Jinan, Shandong 250000 China; 3 Department of proctology, Jinan People’s Hospital, Jinan, Shandong 271100 China; 4 Department of General Surgery, Shandong Provincial Qianfoshan Hospital, Weifang Medical College, Weifang, Shandong 261000 China; 5 Department of Hepatobiliary Surgery, General Surgery, Qilu Hospital of Shandong University, Jinan, Shandong 250000 China; 6 School of Traditional Chinese Medicine, Shandong University of Traditional Chinese Medicine, Jinan, Shandong 250000 China; 7 Medical Research Center, The First Affiliated Hospital of Shandong First Medical University & Shandong Provincial Qianfoshan Hospital, Jinan, Shandong 250000 China

**Keywords:** Cancer stem cells, Stem cells

## Abstract

Gastric cancer stem cells (GCSCs) play critical roles in gastric cancer (GC) initiation and development. Circular RNAs (circRNAs) participate in diverse cancer biological processes and function as tumor suppressors or oncogenes. This study aims to discover the expression profile and functional roles of circRNAs in GCSCs. A spheroid formation assay was conducted to enrich GCSCs. Genome-wide sequencing of circRNAs showed that a novel circRNA, circSLC4A7, was one of the most upregulated circRNAs in GCSCs. CircSLC4A7 was localized to the nucleus, and its level was elevated in GC cells and tissues. Furthermore, circSLC4A7 increased CSC-like properties and drove cell proliferation, migration, and invasion, which were determined by gain- and loss-of-function experiments. Specific circRNA pull-down assays followed by mass spectrometry analysis, RNA immunoprecipitation, and dual RNA-fluorescence in situ hybridization and immunofluorescence assay were conducted and HSP90 was detected to interact with circSLC4A7 and mediate the oncogenic function of circSLC4A7 by activating the Notch1 signaling pathway in GC. This study highlights a novel oncogenic function of circSLC4A7 mediated by its binding with HSP90 and thus activating the Notch1 signaling pathway.

## Introduction

Gastric cancer (GC) ranks as the fifth most commonly diagnosed cancer and the fourth leading cause of cancer-related death worldwide [1]. Patients with GC are usually diagnosed at an advanced stage because of the lack of typical symptoms and effective biomarkers for early stage diagnosis. The overall 5-year survival rate remains low despite tremendous advancements in surgery, chemoradiotherapy and immunotherapy [2]. In addition, the underlying molecular mechanisms of GC are not clear. The limited understanding of mechanisms underlying the initiating and development of GC suggests the need for further investigation. Cancer stem cells (CSCs) constitute a subset of cells with the capacity for self-renewal and differentiation and that contribute to cancer initiation, long-term tumor growth, metastasis and therapeutic resistance in various types of cancers, including GC [3–5]. Therefore, discovering the underlying mechanisms regulating gastric CSC-like properties may improve the understanding of the mechanisms in developing GC.

Circular RNAs (circRNAs) constitute a class of single-stranded closed RNAs that undergo specific backsplicing at the pre-mRNA stage [6]. Recent evidence has shown that circRNAs exist in a variety of human cells and tissues [7]. A functional analysis revealed that circRNAs can serve as either oncogenic stimuli or tumor suppressors in cancer by sponging microRNAs, regulating transcription and splicing, binding and sequestering proteins, and translating proteins [8, 9]. In addition, circRNAs exhibit tissue- and cancer-specific expression patterns and can be enriched and stable in extracellular fluid, making circRNAs ideal therapeutic targets and biomarkers for the early diagnosis and prediction of a prognosis [10]. Some circRNAs, such as circCPVT1, circHECTD1, and circNRIP1, have been shown to participate in the progression and drug resistance of GC [11]. In addition, certain circRNAs displayed relatively high sensitivities and specificities, which may be useful in the diagnosis and prediction of a prognosis in GC [12].

CircRNAs can affect the CSC phenotype via certain classical signaling pathways, including the Wnt/β-catenin signaling pathway [13], HIF1AN/Notch signaling pathway [14], JAK2/STAT3 signaling pathway [15], and Hedgehog signaling pathway [16]. Other studies indicated that circRNAs may attenuate the therapeutic resistance of CSCs or act as potential vaccines in CSC-targeted therapies [17, 18]. Therefore, further exploration into the functional role of circRNAs in CSCs may lead to new directions in cancer treatment. Xia et al. [19] reported that circFAM73A enhanced CSC-like properties and led to GC progression. However, the expression profiles of circRNAs in gastric CSCs (GCSCs) have not been reported, and the detailed mechanisms of circRNAs regulation of stemness in GC deserve further investigation.

Methods to identify CSCs include tumor formation in immunodeficient mice, spheroid formation in vitro, and the detection of the expression of certain cell surface markers. In this study, spheroid formation in vitro was performed to increase the number of GCSCs, and the expression profiles of circRNAs in GCSCs and GC cells were determined by circRNA sequencing. Differentially expressed circRNAs were identified, and circSLC4A7 was chosen for further analysis of its functional role and regulatory mechanisms.

## Materials and methods

### Cell culture and transfection

The GES-1 normal human gastric epithelial cell line and multiple GC cell lines (the AGS, BGC823, MGC803, and SGC7901 cell lines) recently authenticated were purchased from Cell Bank of Type Culture Collection of Chinese Academy of Sciences, Shanghai Institute of Cell Biology, Chinese Academy of Sciences. The cell lines were cultured in DMEM (Gibco, Carlsbad, CA, USA) supplemented with 10% heat-inactivated fetal bovine serum (FBS) (Gibco), 100 U/ml of penicillin, and 100 μg/ml of streptomycin (HyClone) and maintained in a humidified incubator with 5% CO_2_. All the cells were used for this study within 6 months of purchase.

An siRNA against circSLC4A7 was synthesized by GenePharma (Shanghai, China), and targeted to the junction region of the circSLC4A7 sequence. Lipofectamine 3000 (Invitrogen) was used for transfection following the manufacturer’s instructions. The sequence of the siRNA was 5′-AAUAUAGGUAUUUUGGCCUTT-3′, and that of the negative control was 5′-UUCUCCGAACGUGUCACGUTT-3′.

### Patient tissue specimens

A total of 64 GC tissues and corresponding tumor adjacent tissues were obtained from GC patients who underwent radical gastrectomy at the Department of Gastrointestinal Surgery, the First Affiliated Hospital of Shandong First Medical University (Shandong Provincial Qianfoshan Hospital) between February 2018 and February 2022. This study was approved by the Ethics Committees of the First Affiliated Hospital of Shandong First Medical University, and conducted in accordance with the Helsinki Declaration. Each patient signed an informed consent form.

### RNase R treatment

For RNase R treatment, total RNA was incubated for 15 min at 37 °C with or without 3 U/mg RNase R (Epicentre Technologies, Madison, WI, USA) to remove linear RNA. Quantitative real-time polymerase chain reaction (qRT-PCR) was performed to measure the expression of circSLC4A7 and SLC4A7 mRNA.

### Actinomycin D assay

Cells were seeded at 1 × 10^5^ cells per well in a 6-well plate and treated with 3 mg/L actinomycin D (Sigma, USA) for 6, 12 and 18 h. The cells were harvested at the indicated time points and total RNA was collected. The stability of circSLC4A7 was assessed via qRT-PCR.

### Spheroid formation assay

GC cells were seeded into Costar Ultra Low-Cluster 24-well plates (Corning, NY, USA) at a density of 10,000 cells/ml and cultured in DMEM/F12 medium (Sigma) supplemented with 5 ng/ml epidermal growth factor (EGF, Sigma), B27 supplement (1X, Invitrogen), 20 ng/mL basic fibroblast growth factor (bFGF, PeproTech), and 10 ng/mL hepatocyte growth factor (HGF, PeproTech) at 37 °C with 5% CO_2_ and 100% humidity. After incubation for 7 days, the formed spheres were counted. To passage tumor spheres, spheres cultured for 7 days were collected, disaggregated with 0.05% trypsin/EDTA (Solarbio), sieved through a 40-μm filter and seeded as described above.

### RNA pull-down and mass spectrometry

An RNA pull-down assay for investigating RNA-protein interactions was performed using a PureBinding^TM^ RNA-Protein pull-down kit (P0201, GENESEED, Guangdong, China). First, cell samples (1 × 10^7^) were treated with 1 mL of capture buffer mixed with 10 μL of RNase and 10 μL of protease inhibitor. The supernatant was obtained via centrifugation at 14,000 × *g* for 10 min at 4 °C. To prepare probe-coated beads, a biotinylated circSLC4A7 probe or oligo probe (Gene-Pharma, China) was incubated with 50 μL of streptavidin magnetic beads. Then, 50 pmol probe was added to the beads, and the mixture was incubated at 4 °C with rotation for 30 min. The beads were collected after washing twice with 0.5 mL of capture buffer. Subsequently, the probe-coated beads were mixed with 450 μL of supernatant at 4 °C with rotation for 1 h. Next, the beads were collected and washed with 1 mL of wash buffer mixed with 1 μL of RNase and 1 μL of protease inhibitor 3 times. Finally, the mixture was loaded with 40 μL of capture buffer and 40 μL of loading buffer for subsequent examinations. The complex pulled-down was subjected to SDS-PAGE and analyzed by mass spectrometry.

The sequence of the negative control probe for the RNA pull-down assay was 5′-GCUUAACAUGUAUCUUAUUCGA-3′, and the sequence of the circSLC4A7 probe was 5′-AAUACCUAUAUUUUAGGGCCUU-3′.

### RNA-immunoprecipitation (RIP) assay

RIP assays were performed with a Magna RIP RNA-Binding Protein Immunoprecipitation Kit (Millipore, Billerica, MA, USA) according to the manufacturer’s protocol. Briefly, 1 × 10^7^ cells were lysed in RIP lysis buffer containing protease and RNase inhibitors (Millipore). Then, the cell lysates were incubated with antibodies of interest or a nonspecific IgG antibody (Abcam) at 4 °C overnight. Next, the beads were washed with RIP wash buffer, followed by proteinase K digestion at 37 °C for 30 min. Finally, total RNA was isolated from the aqueous solution after digestion and circSCL4A7 enrichment was measured by qRT-PCR.

### Statistical analysis

Results are shown as mean ± standard deviation (SD). Comparisons between different groups were performed using the Student two-tailed t test. ANOVA (analysis of variance) analysis was used for analysis of differences among three or more groups and post-hoc analysis was performed. Multiple comparisons were done after homogeneity test for variance. Variance was similar between the groups that are being statistically compared. *P* < 0.05 was regarded as statistically significant. Statistical analysis was carried out with SPSS 22.0 software (SPSS Inc.).

## Results

### CircRNA expression spectrum in CSCs and MSCs

**Fig. 1:**
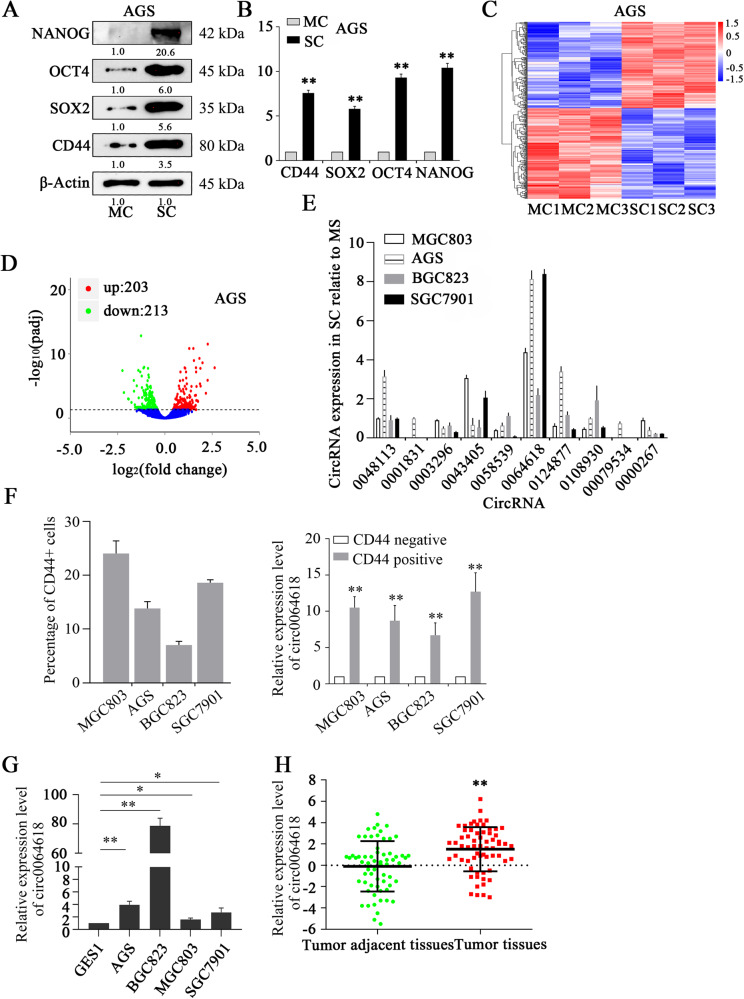
The exclusive circRNA expression spectrum in gastric cancer stem cells. Protein levels of cancer stem cell markers (NANOG, OCT4, SOX2, and CD44) in MCs and SCs as measured by Western blotting (**A**) and qRT-PCR (**B**). **C** Differentially expressed circRNAs determined from circRNA-seq data of 3 groups of MCs and 3 groups of SCs from AGS cells. **D**. Volcano plot of the differentially expressed circRNAs in the circRNA-seq assay. **E** Verification of the expression of the 6 most upregulated circRNAs and the 4 most downregulated circRNAs in SCs (MGC803, AGS, BGC823, and SGC7901 cells) relative to the respective control MCs, as determined by qRT-PCR. **F** The percentages of CD44 positive cells in MGC803, AGS, BGC823, and SGC7901 cells were analyzed by FACS. Expression of circ0064618 in CD44 positive cells versus CD44 negative cells separated from MGC803, AGS, BGC823, and SGC7901 cells as measured by qRT-PCR. **G** Measurement of circ0064618 in GES1 and four gastric cancer cell lines (the MGC803, AGS, BGC823, and SGC7901 lines). **H** Expression of circ0064618 in tumor adjacent tissues and tumor tissues as measured by qRT-PCR. MC, monolayer gastric cancer cells; SC, spheroid gastric cancer stem cells. **P < 0.01 based on the Student *t* test. All results exhibited in (**A**–**H**) (except for **C** and **D**) are from three independent experiments. Data are represented as mean ± SD.

The spheriod formation assay is one of the commonly used methods for enriching CSCs [20]. Our study first verified the overexpression of CSC markers in cells obtained after the spheroid formation assay (spherical cells, SCs), and the marker abundance was compared with that of monolayer cells (MCs) at the mRNA and protein levels (Fig. 1A, B). The expression pattern of circRNAs in GCSCs remained unclear. Therefore, we extracted total RNA from 3 pairs of GC SCs and MCs, and second-generation sequencing was performed to identify differentially expressed circRNAs (Fig. 1C). A gene-cluster analysis revealed that 203 circRNAs were upregulated and that 213 circRNAs were downregulated (Fig. 1D). To verify the results of the gene-cluster analysis, the expression of 6 most upregulated circRNAs and 4 most downregulated circRNAs in 4 GC cell lines (MGC803, AGS, BGC823, and SGC7901) was further detected by qRT-PCR. CircRNA_0064618 (circ0064618) exhibited the highest overexpression level in four SCs compared with MCs (Fig. 1E). CD44 positive GC cells showed an obviously higher expression level of circ0064618 than CD44 negative cells (Fig. 1F). Moreover, the expression of circ0064168 in GES1 cells was much lower than that in GC cell lines (Fig. 1G). Notably, GC tissues exhibited a notably higher expression level of circSLC4A7 than tumor adjacent tissues (Fig. 1H). These results suggested that circ0064618 may play a pivotal role in GCSCs. In addition, AGS and BGC823 cells exhibited higher expression levels of circ0064618 than MGC803 and SGC7901 cells; therefore, circ0064618 was ectopically overexpressed in MGC803 and SGC7901 cells, and silenced in AGS and BGC823 cells to investigate its biological function.

### Specific features and prognostic value of circSLC4A7 in GC cells

**Fig. 2:**
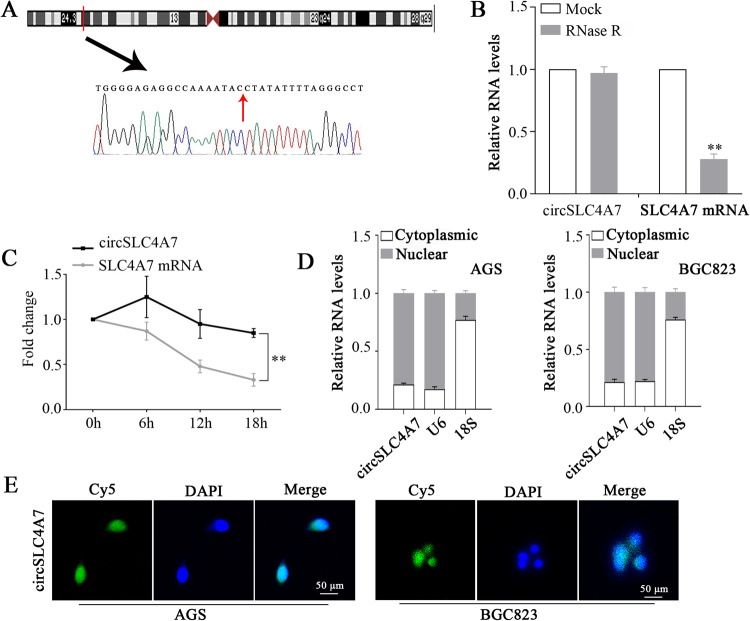
Characterization of circSLC4A7. **A** Scheme illustrating the production of circSLC4A7. **B** Total RNA was digested with RNase R, followed by qRT-PCR detection of circSLC4A7 expression. SLC4A7 mRNA was detected as the RNase R-sensitive control. **C** The relative RNA levels of circSLC4A7 and SLC4A7 were analyzed by RT-qPCR after treatment with actinomycin D at the indicated time points. **D** Cellular localization of circSLC4A7 in AGS and BGC823 cells. The nuclear and cytoplasmic fractions were separated, and the RNA was extracted. CircSLC4A7, U6 and 18 S levels were analyzed by qRT-PCR. The data represent at least three independent experiments. **E** RNA FISH for circSLC4A7 in AGS and BGC823 cells. Nuclei were stained with DAPI. (scale bar, 50 μm). **P < 0.01 based on the Student *t* test. All results are from three independent experiments. Data are represented as mean ± SD. At least one representative image was captured.

Circ0064618 is encoded by the SLC4A7 gene, which is located at chromosome 3 (UCSC data in NCBI, Fig. 2A), and therefore, circ0064618 is termed circSLC4A7. The parent gene of circSLC4A7, SLC4A7, showed a higher expression level in 3 gastric cancer cells (AGS, MGC803, and SGC7901) than in GES1 (Supplementary Fig. 1A). To prevent trans-splicing or genomic rearrangements, including head-to-tail splicing, we employed several universal circRNA detection methods. First, we confirmed head-to-tail splicing in the RT-PCR product of circSLC4A7 that was the expected size by Sanger sequencing. In addition, RNase R, a highly effective 3′ to 5′ exoribonuclease that digests linear RNAs, was used to digest circSLC4A7 and SLC4A7 mRNA, and resistance to RNase R exonuclease confirmed that the RNA species was a circular form (Fig. 2B). Notably, after actinomycin D treatment, circSLC4A7 displayed a significantly longer half-life than SLC4A7 mRNA (Fig. 2C). Further examination with mRNA fractionation (Fig. 2D) and FISH against circSLC4A7 (Fig. 2E) demonstrated that circSLC4A7 was preferentially localized in the nucleus.

### CircSLC4A7 increases the malignant behavior of GC by modulating cell stemness

**Fig. 3:**
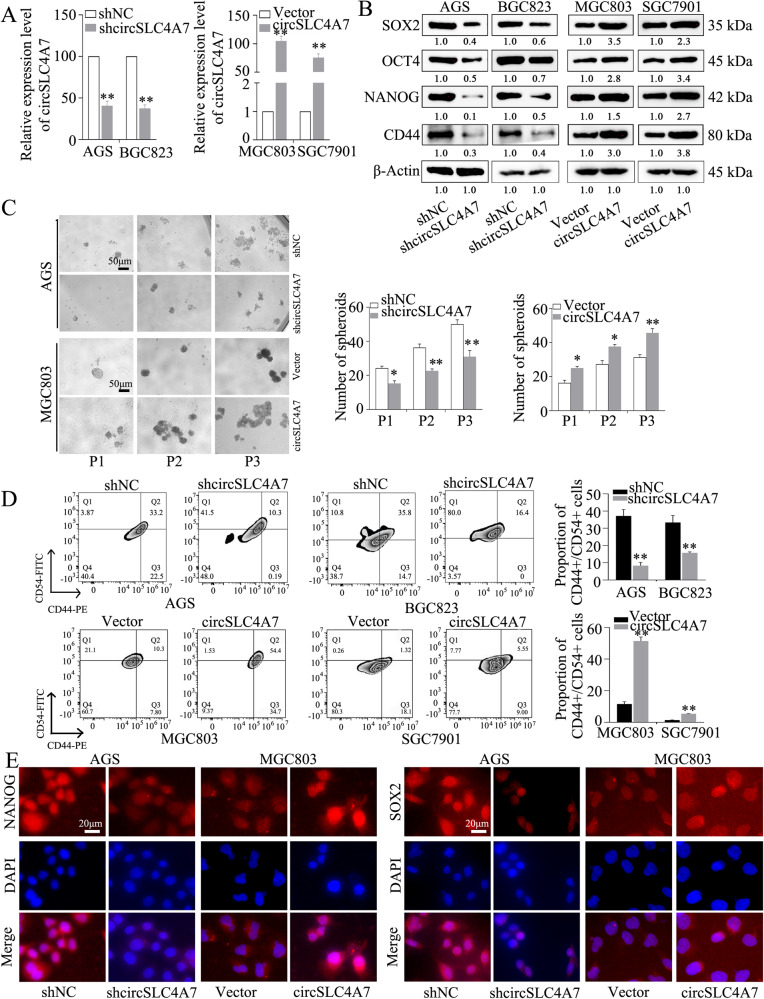
The expression of circSLC4A7 positively correlates with stemness in GC. **A** Relative expression of circSLC4A7 after its silencing in AGS and BGC823 cells and its overexpression in MGC803 and SGC7901 cells. **B** Expression of cancer stem cell markers (NANOG, OCT4, SOX2, and CD44) after silencing circSLC4A7 in AGS and BGC823 cells and overexpressing circSLC4A7 in MGC803 and SGC7901 cells, as measured by Western blotting. **C** Representative images of spheroids formed by AGS-shcircSLC4A7/AGS-Vector cells and MGC803-circSLC4A7/MGC803-Vector cells in suspension cultures. (scale bar, 50 μm). The data are expressed as the number of tumor spheres per well. **D** Flow Cytometry was adopted to detect the proportions of CD44+/CD54+ cells in AGS, BGC823, MGC803, and SGC7901 cells. The data represent at least three independent experiments. **E** An immunofluence assay was conducted to detect the expression of cancer stem markers (NANOG and SOX2) after circSLC4A7 knockdown in AGS cells and after overexpression in MGC803 cells. **P < 0.01 based on the Student *t* test. All results are from three independent experiments. Data are represented as mean ± SD. At least one representative image was captured.

We investigated the precise function of circSLC4A7 in GC. After stable transfection with circSLC4A7 shRNA, the expression of circSLC4A7 was greatly reduced in AGS and BGC823 cells, and after transfection with an overexpression vector, the expression of circSLC4A7 was significantly upregulated in MGC803 and SGC7901 cells, as determined by qRT-PCR (Fig. 3A). Our results showed that the expression of circSLC4A7 was notably increased in GCSCs; therefore, we investigated whether circSLC4A7 exerts an effect on stem cell potential. Silencing of circSLC4A7 in AGS and BGC823 cells obviously inhibited the expression of CSC markers (SOX2, OCT4, NANOG, and CD44), as detected by Western blotting. Moreover, circSLC4A7 overexpression increased the expression of CSC markers (Fig. 3B). In addition, knocking down circSLC4A7 notably decreased the number of tumor spheres in sequential spheroid formation assay in both AGS and BGC823 cells, and ectopic overexpression of circSLC4A7 increased the number of tumor spheres formed (Fig. 3C and Supplementary Fig. 1B, C). And flow cytometry assay confirmed the decrease of the proportion of CD44+/CD54+ cells in cells with circSLC4A7 silence, and the increase of the proportion of CD44+/CD54+ cells in cells with circSLC4A7 overexpression (Fig. 3D). IF assay further demonstrated the downregulation of NANOG and SOX2 in AGS-shcircSLC4A7 cells and BGC823-shcircSLC4A7 cells relative to their expression in control cells (Fig. 3E and Supplementary Fig. 1D), and the upregulation of NANOG and SOX2 in MGC803-circSLC4A7 cells and SGC7901-circSLC4A7 cells compared with control cells (Fig. 3E and Supplementary Fig. 1C). Collectively, these results suggested that circSLC4A7 increased the CSC capacity in GC cells.

**Fig. 4:**
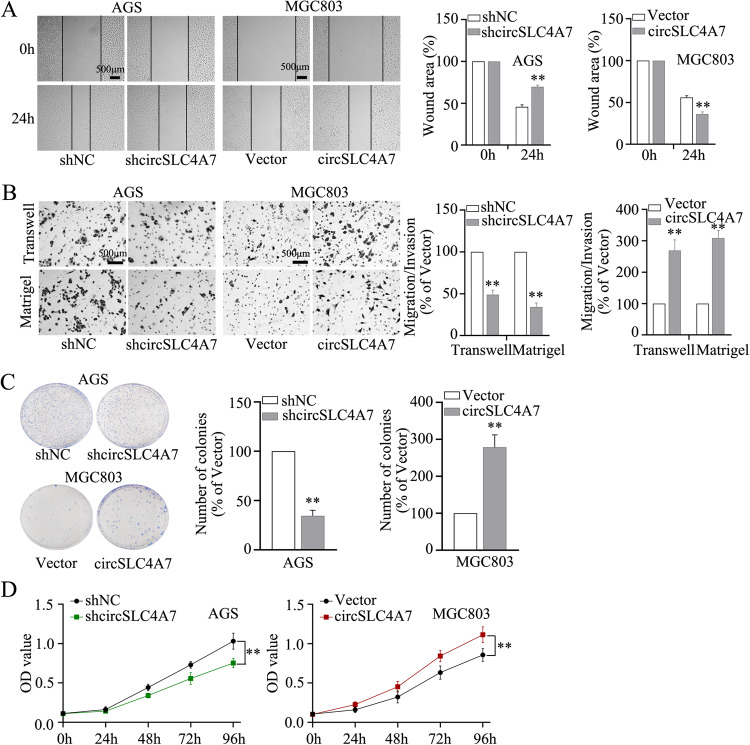
CircSLC4A7 accelerated migration, invasion and proliferation in GC cells. **A** Wound healing assay for AGS-shcircSLC4A7/AGS-Vector cells and MGC803circSLC4A7/MGC803-Vector cells. Representative microscopic images taken 0 and 24 h after wounding are shown on the left (scale bar, 500 μm). The data are expressed as the filled wound area (%). **B** Transwell assay (upper panel) and Matrigel assay (lower panel) with AGS-shcircSLC4A7/AGS-Vector cells and MGC803circSLC4A7/MGC803-Vector cells. The data show the migrated/invaded cells (% of the vector). **C** Colony formation assay for AGS-shcircSLC4A7/AGS-Vector cells and MGC803circSLC4A7/MGC803-Vector cells. The data are displayed as the number of colonies formed (% of Vector). **D** Cell counting kit-8 assays were performed to assess the proliferation of AGS-shcircSLC4A7/AGS-Vector cells and MGC803circSLC4A7/MGC803-Vector cells. **P < 0.01 based on the Student *t* test. All results are from three independent experiments. Data are represented as mean ± SD. At least one representative image was captured.

To detect the role of circSLC4A7 in CSC-related behaviors, the metastatic and proliferative abilities of GC cells were investigated. A wounding healing assay showed that knocking down circSLC4A7 markedly impeded the migration of GC cells and that ectopic expression of circSLC4A7 promoted the migration of GC cells (Fig. 4A and Supplementary Fig. 2A). These results were further confirmed by Transwell assay (Fig. 4B and Supplementary Fig. 2B). In addition, we proved that inhibition of circSLC4A7 markedly retarded cell proliferation and overexpression of circSLC4A7 accelerated cell proliferation in colony formation and CCK-8 assays (Fig. 4C, D and Supplementary Fig. 2C, D). Taken together, these findings revealed that circSLC4A7 promoted migration, invasion and proliferation in GC.

### CircSLC4A7 functions by interacting with HSP90 in GC

**Fig. 5:**
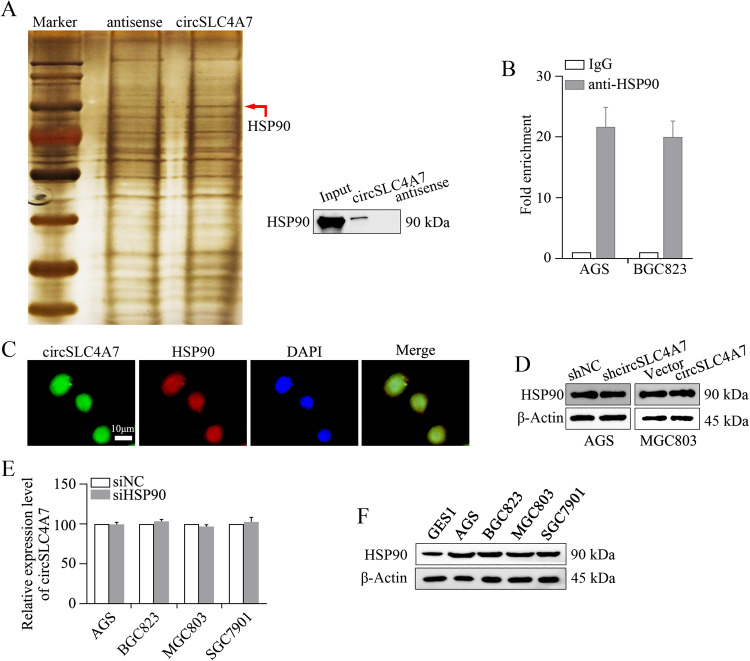
circSLC4A7 interacts with HSP90. **A** RNA pull-down and mass spectrometry assays were used to identify the proteins interacting with circSLC4A7. **B** The interaction between circSLC4A7 and HSP90 was determined by RIP assay. **C** Colocalization of circSLC4A7 and HSP90 detected by FISH and IF. Nuclei were stained with DAPI. **D** Expression of HSP90 was measured in AGS-shcircSLC4A7/AGS-Vector cells and MGC803circSLC4A7/MGC803-Vector cells by Western blot assay. **E** Relative expression level of circSLC4A7 was measured in AGS, BGC823, MGC803, and SGC7901 cells transfected with siNC or siHSP90 by qRT-PCR. **F** Expression of HSP90 was measured in GES1 and 4 gastric cancer cell lines (the MGC803, AGS, BGC823, and SGC7901 cell lines). **P < 0.01 based on the Student *t* test. All results are from three independent experiments. Data are represented as mean ± SD. At least one representative image was captured.

Subsequently, we investigated the mechanism through which circSCL4A7 exerts its function on GC progression. To identify the protein partner of circSLC4A7, we performed biotin-labeled RNA pull-down assay followed by a proteomic analysis of the RNA-associated protein complex in cancer cells. Mass spectrometry assays revealed 169 proteins that may interact with circSLC4A7 in BGC823 cells. The expression of HSP90 was greatly changed (Fig. 5A). The differential expression of HSP90 was further demonstrated by Western blotting (Fig. 5A). In addition, a RIP assay indicated the endogenous enrichment of circSLC4A7 in RNA co-precipitated with an anti-HSP90 antibody in AGS cells and BGC823 cells, demonstrating the interaction between circSLC4A7 and HSP90 (Fig. 5B). In addition, overlapping localization of circSLC4A7 and HSP90 in BGC823 cells was identified by dual RNA-FISH and immunofluorescence assays (Fig. 5C). Furthermore, the expression of HSP90 was not affected after modulating the expression of circSLC4A7 in AGS and MGC803 cells (Fig. 5D). The relative expression levels of circSLC4A7 were also comparable in cells transfected with siHSP90 or siNC (Fig. 5E).The relative expression of HSP90 in GES1 and GC cells (AGS, BGC823, MGC803 and SGC7901 cells) was consistent with that of circSLC4A7 (Fig. 5F). Overall, these results suggested that circSLC4A7 interacted with HSP90 in GC cells.

**Fig. 6:**
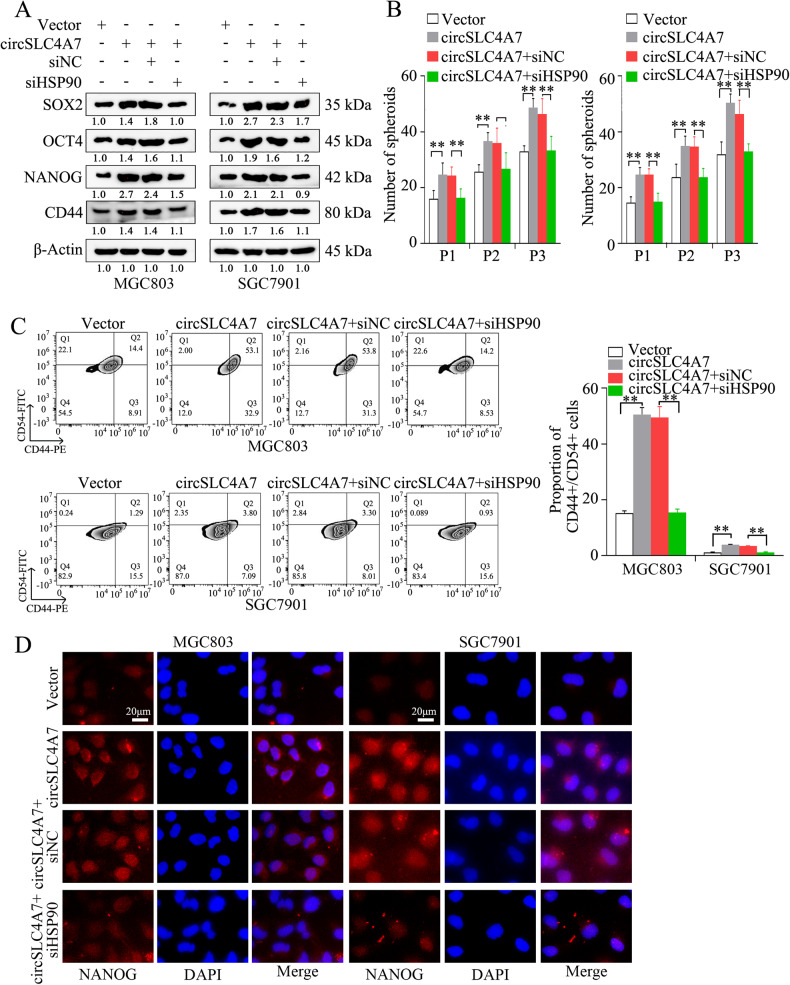
HSP90 mediated the function of circSLC4A7 in regulating stemness. **A** Increased expression of cancer stem cell markers (NANOG, OCT4, SOX2, and CD44) in MGC803-circSLC4A7 cells and SGC7901-circSLC4A7 cells was reversed after transfection with siHSP90. **B** The increase in the number of tumor spheres comprising after MGC803-circSLC4A7 cells and SGC7901-circSLC4A7 cells per well was reduced after the cells were transfected siHSP90. **C** The increased proportions of CD44+/CD54+ cells were reduced after cell transfection with siHSP90. The data represent at least three independent experiments. **D** The upregulated expression of NANOG after circSLC4A7 overexpression in MGC803 and SGC7901 cells was suppressed after cell transfection with siHSP90. (scale bar, 20 μm) **P < 0.01 based on the one-way ANOVA. All results are from three independent experiments. Data are represented as mean ± SD. At least one representative image was captured.

**Fig. 7:**
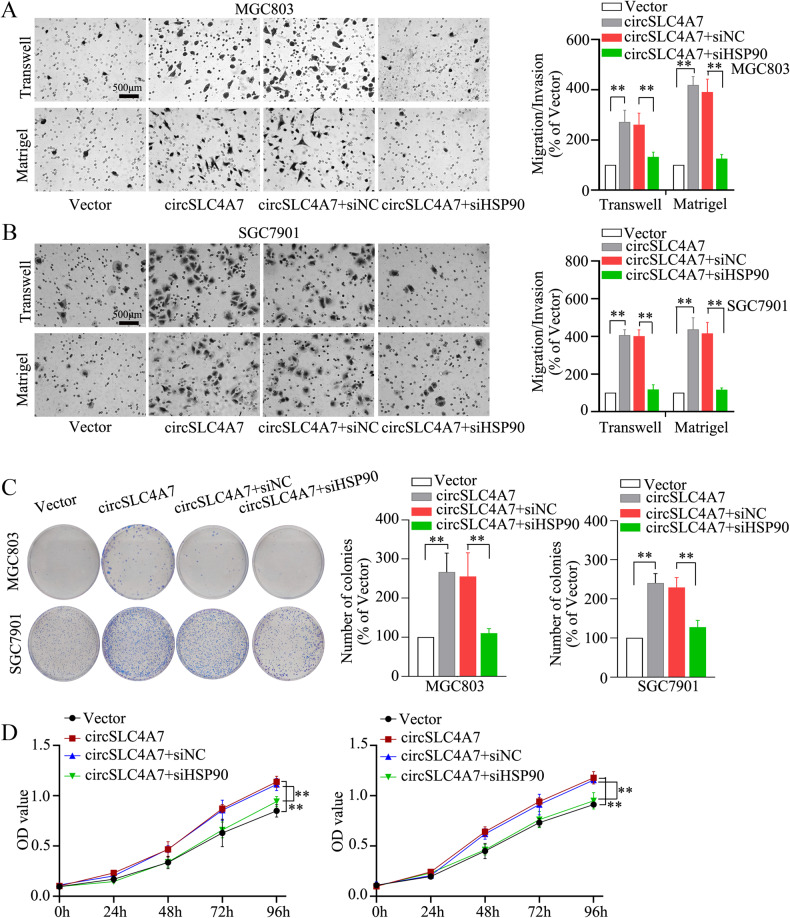
HSP90 mediated the function of circSLC4A7 promoting migration, invasion and proliferation. The increase in the number of migrated/invaded MGC803-circSLC4A7 cells (**A**) and SGC7901-circSLC4A7 cells (**B**) was reduced after the cells were transfected with siHSP90. (scale bar, 500 μm) The increased in the number of colonies (**C**) and OD values (**D**) of MGC803-circSLC4A7 cells and SGC7901-circSLC4A7 cells as reduced after cells were transfected with siHSP90. **P < 0.01 based on the one-way ANOVA. All results are from three independent experiments. Data are represented as mean ± SD. At least one representative image was captured.

The influence of HSP90 expression on the functional role of circSLC4A7 in GC cells was investigated by inhibiting HSP90 expression in circSLC4A7 overexpressing cells (MGC803-circSLC4A7 and SGC7901-circSLC4A7 cells). The results showed that HSP90 silencing profoundly inhibited the upregulation of CSC markers (SOX2, OCT4, NANOG, and CD44), as determined by Western blotting (Fig. 6A). The increase in the number of spheroids formed in the spheroid formation assay was also attenuated (Fig. 6B). In addition, flow cytometry assay showed that siHSP90 reversed the increase of the proportions of CD44+/CD54+ cells caused by circSLC4A7 overexpression (Fig. 6C). IF assays revealed that knocking down HSP90 significantly reduced the increase in the expression of CSC markers (NANOG and SOX2) caused by ectopic circSLC4A7 expression (Fig. 6D and Supplementary Fig. 2E). Moreover, the accelerated migration (Fig. 7A, B, top panel), invasion (Fig. 7A, B, bottom panel), and proliferation (Fig. 7C, D) of circSLC4A7-overexpressing GC cells were also suppressed by silencing HSP90. Overall, this evidence indicated that circSLC4A7 exerted its function by interacting with HSP90 in GC cells.

### CircSLC4A7 binds with HSP90 and modulates cellular stemness in GC through the NOTCH signaling pathway

**Fig. 8:**
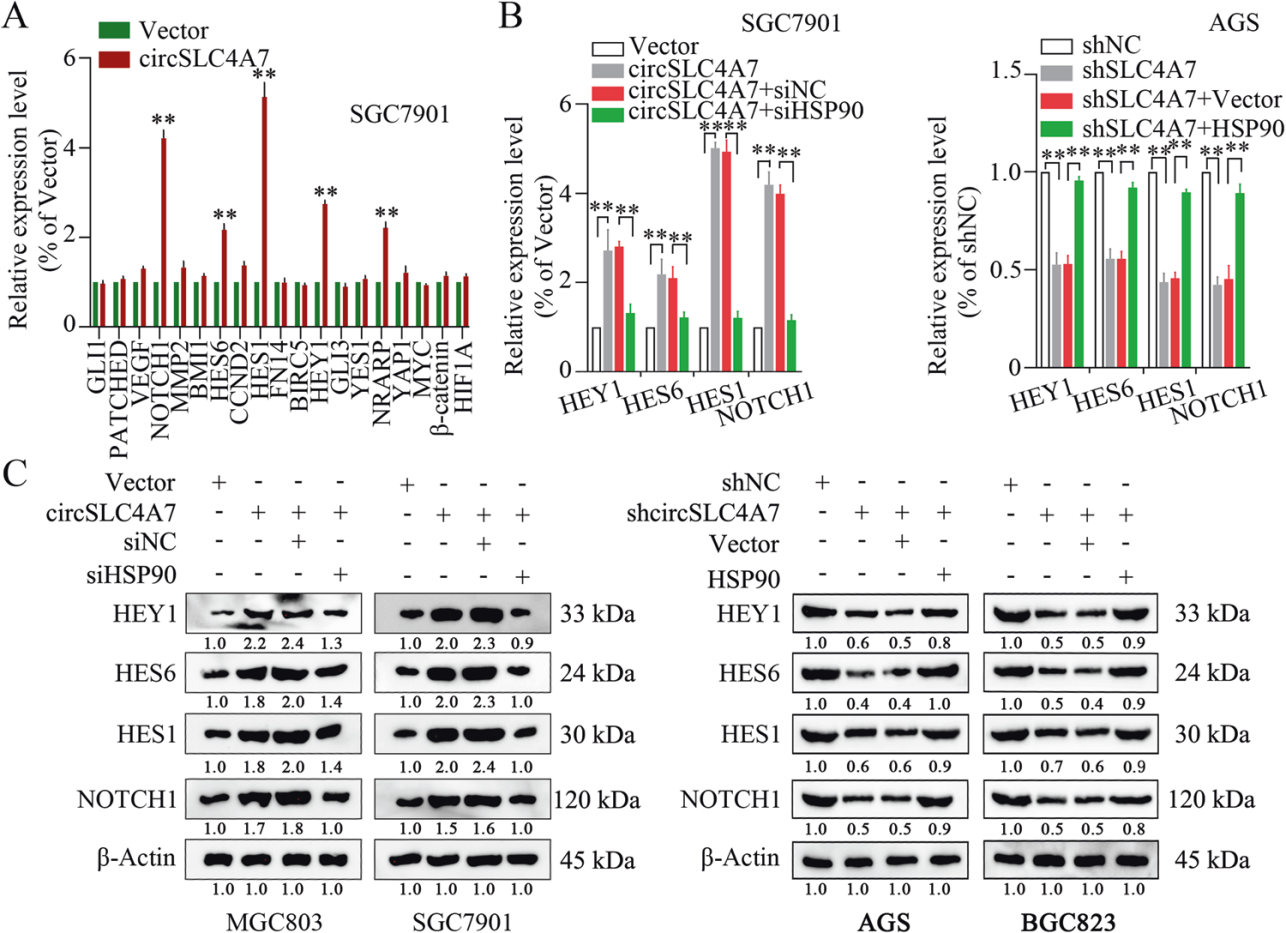
CircSLC4A7 exerts its function through the NOTCH1 signaling pathway by interacting with HSP90. **A** Detection of genes involved in signaling pathways regulating stemness, as measured by qRT-PCR. **B** HSP90 interference reversed the increase of mRNAs levels of NOTCH1 and downstream molecules caused by circSLC4A7 overexpression by qRT-PCR, and HSP90 overexpression reversed the suppression of mRNAs levels of NOTCH1 and downstream molecules caused by circSLC4A7 interference by qRT-PCR. **C** siHSP90 reversed the upregulation of NOTCH1 and downstream molecules caused by the ectopic overexpression of circSLC4A7, overexpression of HSP90 reversed the suppression of NOTCH1 and downstream molecules caused by circSLC4A7 interference. **P < 0.01 based on the one-way ANOVA. All results are from three independent experiments. Data are represented as mean ± SD. At least one representative image was captured.

Several pivotal signaling pathways, including the NOTCH signaling, Wnt signaling, NF-κB signaling and Sonic Hedgehog signaling, play essential roles in modulating CSC characteristics [21]. To identify whether these signaling pathways mediate the function of the circSLC4A7/HSP90 complex in GC, the expression levels of their target genes (NOTCH1, β-catenin, HES6, HEY1, HES1, and NRARP for NOTCH signaling; MYC, CCND2, and FN14 for Wnt signaling; VEGF, HIF1A, BIRC5, MMP2 for NF-κB signaling; BMI1, GLI1, PATCHED, GLI3 for Sonic Hedgehog signaling; YES1 and YAP1 for Hippo-Yap signaling) were measured by qRT-PCR assay. The expression of the genes in the Notch signaling pathway (NOTCH1, HES6, HEY1, HES1, NRARP) were markedly increased in a qRT-PCR assay (Fig. 8A). Further measurements found that HSP90 silencing reversed the upregulated expression of certain genes in the Notch signaling pathway (NOTCH1, HES6, HEY1, and HES1), and HSP90 overexpression reversed the suppression of certain genes in the Notch signaling pathway (NOTCH1, HES6, HEY1, and HES1) on mRNA and protein levels (Fig. 8B, C). In summary, Notch signaling modulated the functional role of the circSLC4A7/HSP90 complex in GC.

## Discussion

Rengganaten et al. [22] conducted genome-wide sequencing of circRNAs specific to colorectal cancer parental and spheroid cells to map the circRNA profile. They verified the stem cell-like properties of the spheroids in spheroid formation assay by analyzing the self-renewal ability, differentiation ability and chemoresistance of colorectal cancer spheroid CSCs. This study mapped the circRNAs profile in GCSCs by sequencing circRNAs in spheroid CSCs; the description of the circRNA profile added a new layer of information onto the GC genome. Our results identified circSLC4A7 as one the most upregulated circRNAs, and tumor tissues showed a profoundly upregulated expression level of circSLC4A7 compared with tumor adjacent tissues. The increased level of circSLC4A7 suggested that this circRNA may play a pivotal functional role in GCSCs. The subsequent observations supported our supposition, confirming that circSLC4A7 endowed cells with significantly increased CSC properties and promoted migration, invasion and proliferation.

The mechanisms by which circRNAs exert their functions are diverse [23]. Some circRNAs can interact with RNA-binding proteins and function as protein sponges or inhibitors; some circRNAs can act as scaffolds to bring different proteins into proximity; and certain other proteins can recruit proteins to specific subcellular compartments [23]. Several circRNAs can function by directly binding to HSP90. For example, circSHKBP1 directly bound to HSP90 and inhibited its ubiquitination [24]. In addition, Bi et al. [25] found that circRNA_102171 affected CTNNBIP1 function but did not influence its expression level [25]. HSP90 was also found to be a binding target of circSTK40, and this bound circRNA formed a scaffold to block the interaction of HSP90 with CLU and thus hindered the proteasomal degradation of HSP90 [26]. In addition, whole-transcriptome sequencing of the Caco-2 colorectal cancer cell line lead to a circRNA-miRNA-mRNA regulatory network, which showed HSP90 inhibitor-induced cell death, implying that numerous circRNAs may function by binding HSP90. The present study revealed that circSLC4A7 functioned by physically interacting with HSP90 without affecting the HSP90 protein level. To discover the detailed mechanism by which circSLC4A7 plays functional roles by binding with HSP90 needs further investigation in future studies.

HSP90 is a highly abundant ubiquitous molecular chaperone that regulates the late-stage maturation, activation, and stability of a diverse range of client proteins [27]. By participating in cell cycle control, cell survival, hormone and other signaling pathways, HSP90 plays an essential role in maintaining cellular homeostasis [28]. Previous studies revealed that the HSP90 level is increased in many solid tumors, and that HSP90 facilitates the function of numerous oncoproteins [29]. HSP90 inhibitors, such as 17-AAG, have been reported to be effective anticancer drugs, and clinical trials are being performed to determine their effectiveness and efficacy [29–33]. In addition, low doses of 17-AAG have been shown to eliminate CSCs in lymphnoma [34]. Combination treatment with 17-AAG and a SIRT1 inhibitor successfully targeted CSCs that are resistant to current therapies in CD44^high^ chronic myeloid leukemia [35]. Similar results were obtained in solid cancers, including thyroid CSCs [36], lung CSCs [37, 38], and breast CSCs [31]. In advanced GC, high expression of HSP90 has been associated with tumor aggressiveness and poor prognosis [39, 40]. Plasma HSP90α has been associated with metastatic stage and showed moderate diagnostic performance in GC [41]. Our study confirmed that circSLC4A7 physically interacted with HSP90. Further observations found that circSLC4A7 cooperated with HSP90 to increase the CSC properties of GC cells and accelerate GC progression. These results need to be verified by in vivo assays; nevertheless, the utilization of HSP90 inhibitors in GC is promising for treating patients resistant to current therapies.

Abnormal activation of Notch signaling is implicated in the self-renewal of various types of CSCs [42], such as head and neck squamous cell carcinoma [43], colorectal cancer [44], hepatocellular carcinoma [45], pancreatic cancer [46], and cholangiocarcinoma [47]. In GC, Notch has been revealed to be a major signaling pathway in mediating CSC functions [48, 49]. In addition, Notch1 directly induces CD133, a well-known CSC biomarker in GC, with diffuse expression in GC [50]. In addition, a meta-analysis of 1547 GC cases and 450 controls revealed that the expression level of Notch1 is an independent poor prognostic predictor in GC. Notably, Wang et al. [51] reported that stabilization of NOTCH1 by the HSP90 chaperone is essential for T-cell leukemogenesis. In our study, both the mRNA and protein levels of NOTCH1 and its downstream proteins, namely, HES1, HEY1, NRARP, and HES6, were notably upregulated in circSLC4A7 overexpressing cells, and this effect was reversed through interference with the expression of HSP90. Changes in the mRNA levels of NOTCH1 and Notch-dependent genes indicated that HSP90 may regulate their expression at the transcriptional level, and further investigation is needed to elucidate the detailed mechanisms. The results confirm that circSLC4A7 regulates GCSC features mediated through the NOTCH1 signaling pathway.

## Conclusion

In conclusion, our research revealed that circSLC4A7 interacted with HSP90 without influencing HSP90 expression level, and then activated the NOTCH1 signaling pathway to regulate GC stemness and progression. These results suggest that circSLC4A7 is a novel promising therapeutic target in GC.

## Supplementary information

Supplemental Materials and Methods

Supplemental Figure Legends

Supplemental Figure 1

Supplemental Figure 2

STable 1

STable 2

Reproducibility checklist

Western blots

## Data Availability

The authors confirm that the data supporting the findings of this study are available within the article or its supplementary materials.
