# Supplementary material for: circSLC4A7 accelerates stemness and progression of gastric cancer by interacting with HSP90 to activate NOTCH1 signaling pathway
Source: Cell Death Dis. 2023 Jul 20;14(7):452. doi: 10.1038/s41419-023-05976-w (PMC10359325; doi:10.1038/s41419-023-05976-w)
Supplement: Supplementary file 1 — Supplemental Materials and Methods [file 41419_2023_5976_MOESM1_ESM.docx]

**Supplemental Materials and methods**

**RNA isolation, reverse transcription and quantitative real-time polymerase chain reaction (qRT-PCR)**

Total RNA from paired tissues or cells were extracted by using TRIzol reagent (Thermo Fisher Scientific, Waltham, MA, USA) following the manufacturer’s instructions. QRT-PCR was performed using SYBR Green and detected using the Applied Biosystems Real-Time PCR System. Relative mRNA expression level was analyzed using the 2^-△△Ct^ method. Glyceraldehyde 3-phosphate dehydrogenase (GAPDH) was used as an internal control. The primers and RNA sequences used in this study are shown in Supplementary Table 1.

**RNA isolation of nuclear and cytoplasmic fractions**

The nuclear and cytoplasmic fractions were extracted using the PARIS™ Kit (Invitrogen) according to the manufacturer’s protocol. Briefly, cells were collected and resuspended in cell fraction buffer. After that, the cells were centrifuged, and the supernatant and lysate were preserved for RNA extraction using a cell disruption buffer, and then mixed with a 2× lysis/binding solution and added equal volume of ethanol. The mixture was washed and eluted for obtaining the cytoplasmic and nuclear RNA.

**Western blot**

Total cellular protein was lysed in RIPA buffer containing complete protease and phosphatase inhibitor cocktail (Sigma, USA). Then, the same amount of protein samples were subjected to SDS–PAGE, transferred to PVDF membrane (Millipore), incubated with the primary antibody overnight at 4 °C, then with the HRP-conjugated secondary antibody for 2 h at room temperature. And the protein bands were exposed by ECL (Millipore, Boston, MA, USA). β-Actin (Cell Signaling Technology) served as control for normalization. The detailed information of the antibodies used in this study can be found in Supplemental Table 2.

**Immunofluorescence Assay**

Cells were seeded on glass coverslips. After overnight incubation, the cells were fixed with 4% formaldehyde for 15 min, and permeabilized in 0.4% Triton X-100 for 15 min at room temperature. The cells were then incubated with 5% BSA for 1 hour at room temperature. Subsequently, they were incubated with primary antibody overnight at 4 °C. Next day, they were incubated with FITC-conjugated secondary antibody for 1 h at 37 °C. DAPI was used to stain cell nuclei, and a Zeiss LSM700 confocal microscope (Carl Zeiss, Oberkochen, Germany) was used for taking pictures.

**Cell viability assay**

Cell proliferation was assessed by using the cell counting kit-8 (CCK-8, Sigma-Aldrich) following the manufacturer’s instructions. Cells were seeded into 96-well plates and incubated at described days in a 5% CO_2_ atmosphere at 37°C. CCK-8 solution (10 μl) was added to each well at indicated time points (0, 24, 48, 72, and 96 h). The absorbance at 490 nM was measured using SpectraMax 250 spectrophotometer (Molecular Devices, USA).

**Colony formation assay**

A total of 500 cells per well were seeded into 6-well plates and incubated for 14 days to form the colonies. Then cells were fixed with methanol and stained with 0.5% crystal violet. The colonies were photographed and counted. All the experiments were performed at least triplicate wells.

**Wound healing assay**

Cells were seeded into 6-well plates and cultured overnight. A straight scratch was made by using a pipette tip. Fresh medium was added, and images of the same location were acquired at 0h and 24 h.

**Transwell and Matrigel assay**

Twenty-four-wells were coated with diluted Matrigel (BD Biosciences) in PBS and dried for 6 h. The cells were resuspended in serum-free media, and then seeded in each top chamber of Transwells (8.0-mm, BD Biosciences). The lower chambers were filled with 10% FBS complete media for chemoattraction. Chambers were incubated for 48 h in a 37 ℃ incubator supplemented with 5% CO_2_. Non-invaded cells on the upper surface were removed with a cotton swab. and the cells on the lower surface were fixed in 4% paraformaldehyde for 30 min, and stained with crystal violet solution for 10 min. Images were captured under a microscope.

For the Matrigel assay, cells were seeded onto the Matrigel (BD Biosciences, San Jose, CA, USA) precoated in the transwell chamber, the following procedures were conducted as the Transwell assay.

**Fluorescence in situ hybridization (FISH)**

The FISH probe for circSLC4A7 was designed and synthesized by Genepharma (Shanghai, China). RNA FISH kit was purchased from Genepharma. Briefly, cells were evenly inoculated in a 24-well plate, and the cells were washed, fixed, permeabilized, and prehybridized. Then, the cells were incubated with circSLC4A7 probe at 37 °C overnight, and rinsed in SSC buffer at 42 °C. Then nuclear staining was performed with DAPI, followed by fluorescence microscopy. The sequence of circSLC4A7 probe for FISH was 5’-AAAATACCTATATTTTAGGGCCTTG-3’.
