# Supplementary material for: circSLC4A7 accelerates stemness and progression of gastric cancer by interacting with HSP90 to activate NOTCH1 signaling pathway
Source: Cell Death Dis. 2023 Jul 20;14(7):452. doi: 10.1038/s41419-023-05976-w (PMC10359325; doi:10.1038/s41419-023-05976-w)
Supplement: Supplementary file 2 — Supplemental Figure Legends [file 41419_2023_5976_MOESM2_ESM.docx]

Supplemental Figure 1. The expression of circSLC4A7 positively correlates with stemness in GC. A. Expression of the parent gene for circSL4A7, SLC4A7, in gastric cancer cell lines and GES1. B and C. Representative images of spheroids formed by BGC823-shcircSLC4A7/BGC823-Vector cells (B) and SGC7901-circSLC4A7/SGC7901-Vector cells (C) in suspension cultures. (scale bar, 50 μm). The data are expressed as the number of tumor spheres per well. D. An immunofluence assay was conducted to detect the expression of cancer stem markers (NANOG and SOX2) after circSLC4A7 knockdown in BGC823 cells and overexpressed in SGC7901 cells. ** *P* < 0.01 based on the Student *t* test. All results are from three independent experiments. Data are represented as mean ± SD. At least one representative image was captured.

Supplemental Figure 2. circSLC4A7 promoted the migration, invasion and proliferation of GC cells. A. Wound healing assays for BGC823-shcircSLC4A7/ BGC823-Vector cells and SGC7901-circSLC4A7/SGC7901-Vector cells. Representative microscopy images taken 0 and 24 hours after wounding are shown on the left (scale bar, 500 μm). The data are expressed as the filled wound area (%). B. Transwell assay (upper panel) and Matrigel assay (lower panel) for BGC823-shcircSLC4A7/ BGC823-Vector cells and SGC7901-circSLC4A7/SGC7901-Vector cells. The data are shown as Migrated/Invaded cells (% of vector) C. Colony formation assay with BGC823-shcircSLC4A7/ BGC823-Vector cells and SGC7901-circSLC4A7/SGC7901-Vector cells. The data are displayed as the number of colonies formed (% of Vector). D. Cell counting kit-8 assays were performed to assess for the proliferation of BGC823-shcircSLC4A7/ BGC823-Vector cells and SGC7901-circSLC4A7/SGC7901-Vector cells. E. The upregulated expression of SOX2 caused by circSLC4A7 overexpression in MGC803 and SGC7901 was suppressed after the cells were transfected with siHSP90. ** *P* < 0.01 based on the Student *t* test. All results are from three independent experiments. Data are represented as mean ± SD. At least one representative image was captured.
