# Supplementary figures and images for: circSLC4A7 accelerates stemness and progression of gastric cancer by interacting with HSP90 to activate NOTCH1 signaling pathway
Source: Cell Death Dis. 2023 Jul 20;14(7):452. doi: 10.1038/s41419-023-05976-w (PMC10359325; doi:10.1038/s41419-023-05976-w)

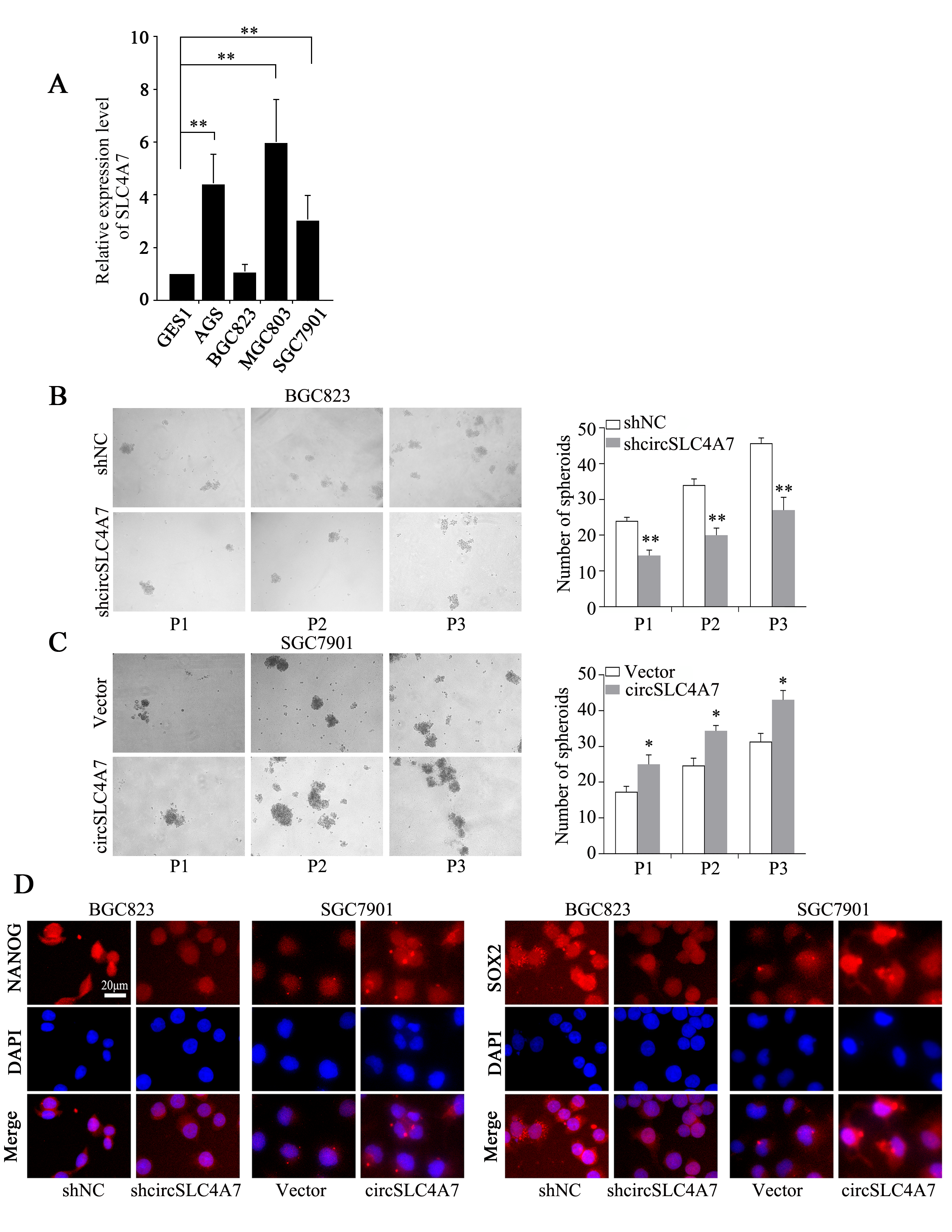

Supplement: Supplementary file 3 — Supplemental Figure 1 [file 41419_2023_5976_MOESM3_ESM.tif]

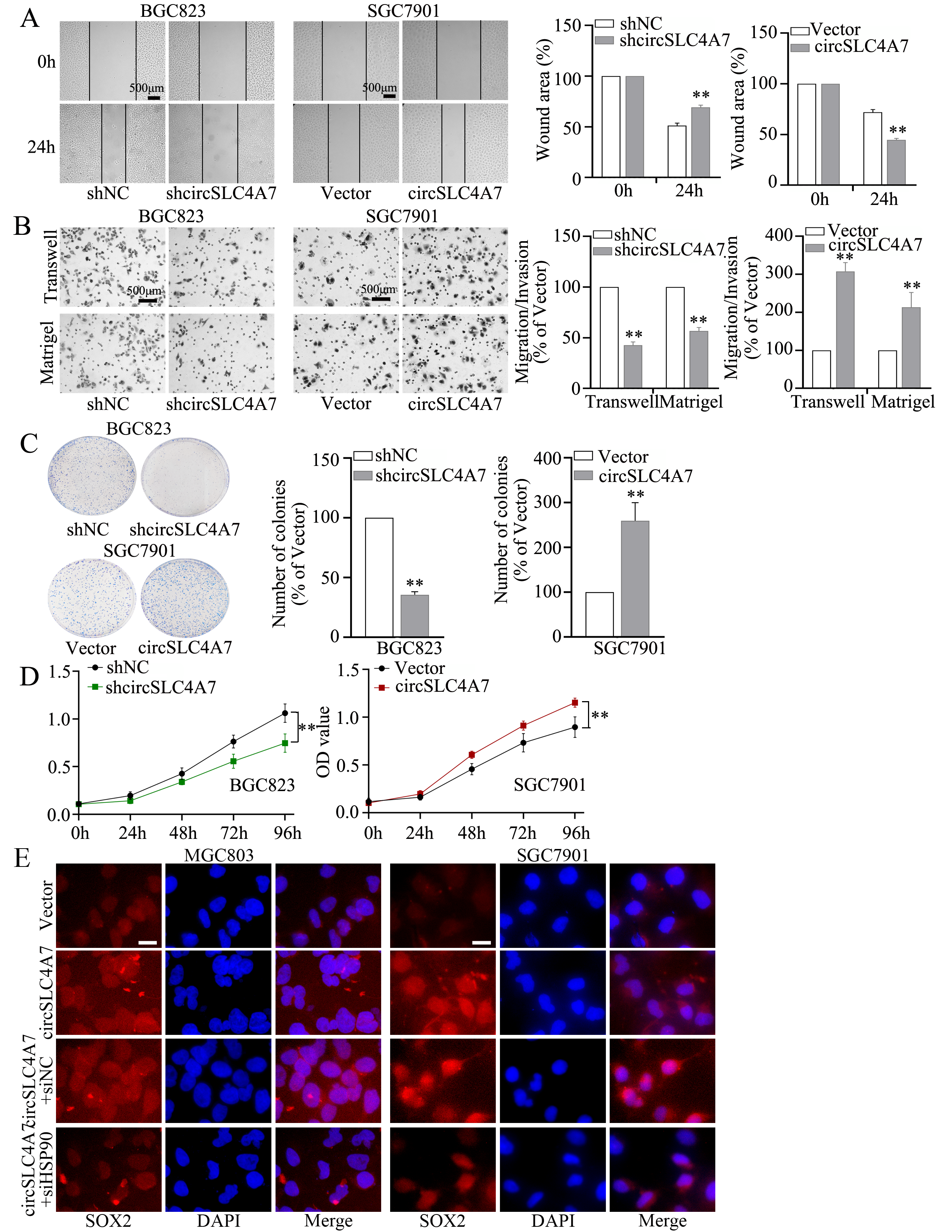

Supplement: Supplementary file 4 — Supplemental Figure 2 [file 41419_2023_5976_MOESM4_ESM.tif]

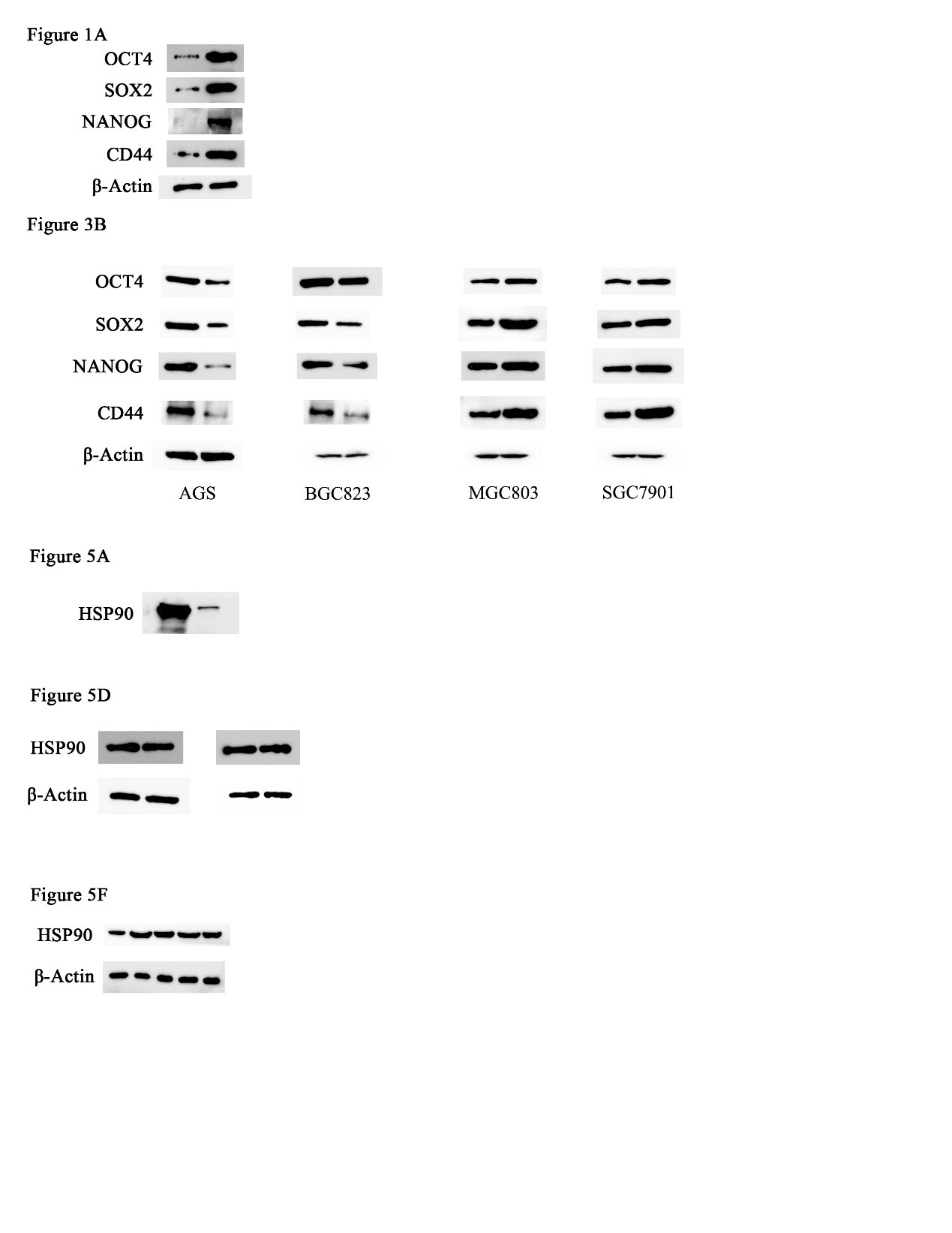


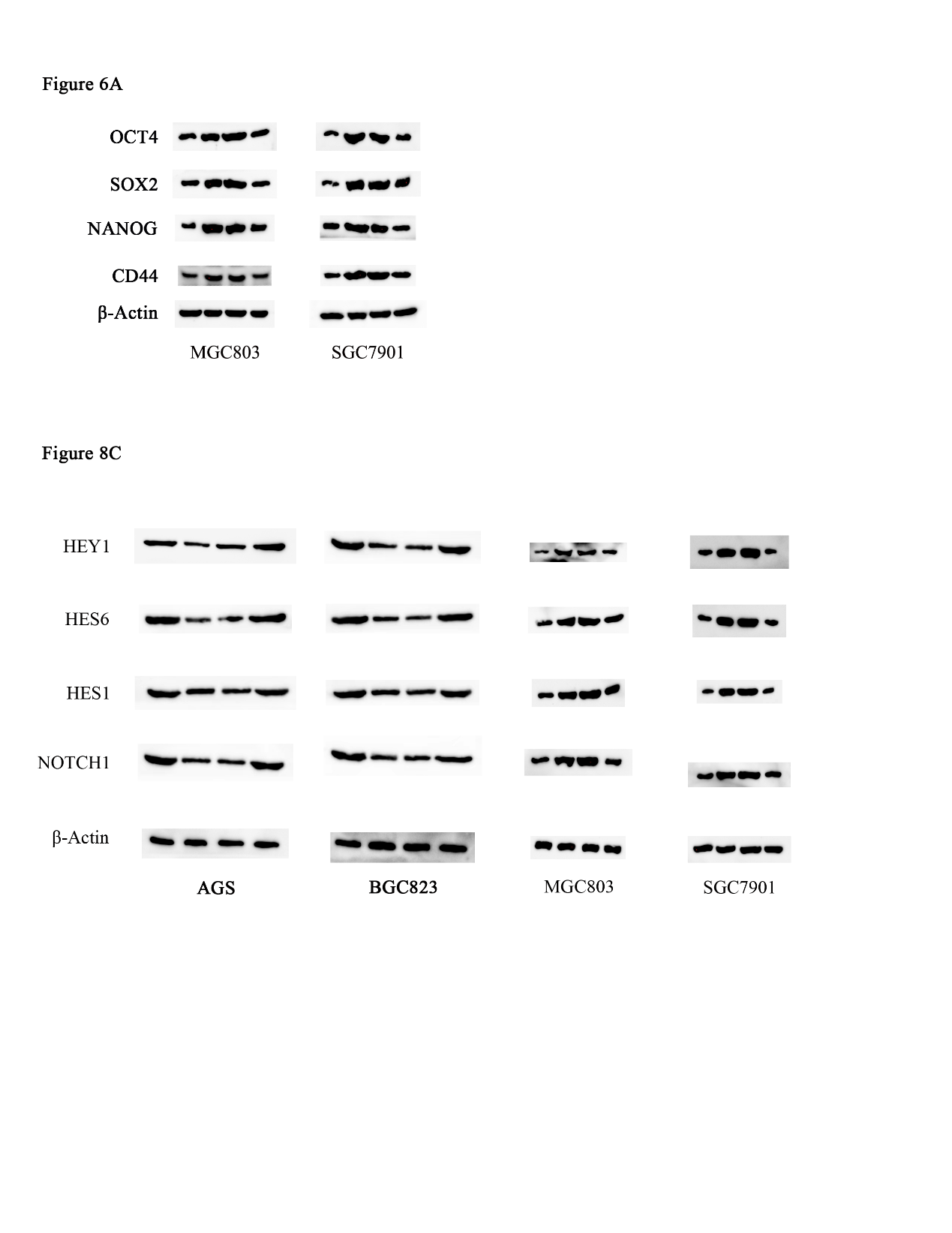

Supplement: Supplementary file 8 — Western blots [file 41419_2023_5976_MOESM8_ESM.docx]
