# Supplementary material for: circSLC4A7 accelerates stemness and progression of gastric cancer by interacting with HSP90 to activate NOTCH1 signaling pathway
Source: Cell Death Dis. 2023 Jul 20;14(7):452. doi: 10.1038/s41419-023-05976-w (PMC10359325; doi:10.1038/s41419-023-05976-w)
Supplement: Supplementary file 5 — STable 1 [file 41419_2023_5976_MOESM5_ESM.docx]

| Name | Forward sequence | Reverse sequence |
| --- | --- | --- |
| CD44 | TGGAGAAAAATGGTCGCTACAG | GGGCAAGGTGCTATTGAAAGC |
| SOX2 | GCCGAGTGGAAACTTTTGTCG | GGCAGCGTGTACTTATCCTTCT |
| OCT4 | GGGAGATTGATAACTGGTGTGTT | GTGTATATCCCAGGGTGATCCTC |
| NANOG | ATGCCTGTGATTTGTGGGCC | GCCAGTTGTTTTTCTGCCAC |
| SLC4A7 | ACAGAAGGCAGAATAAGTGCAATAGA | AGGTTGCCCAGCAAACAATG |
| circ_0043405 | AGCTCCATGAGAAGACGGAAGA | CATCTTCCTGACCCTCAAATTCC |
| circ_0003296 | ACCAGACATAGCCCAAAGAGC | TCAGGAGCCATCCAACATGG |
| circ_0001831 | GGAACGGCACAGAGTGTTTG | AAAGGGTGTCATCGGAGAGC |
| circ_0048113 | CGCCGTCCACTTAAAGGCTA | CAGATCGTTGGTGGCCGATA |
| circ_0000267 | AGAAGTGCAAGAGCAGAGGC | TGTCCTTTTCAGGCAGGCAT |
| circ_0124877 | GAGGGTGCATCAATTCTTCGAC | CTGTAAGCCGGTGACTTGGA |
| circ_0108930 | TGCTGGATATTGATGCGCCAG | CCAGCCTTCCCTCCTTCATA |
| circ_0064618 | TCCATTTGAAGGGGGAAGTCTC | GCTTCCACCACTTCCATTACC |
| circ_0058539 | ACTGGTAGCCCAAGATTGCC | AGATGACTGAGTGGGAAGACTG |
| circ_0079534 | CACAAAGCAACAAATGGAGGC | TGTGCAATTCTTCCTGACCG |
| HES6 | TGACCACAGCCCAAATTGCC | TCAGAGGAGGGAGGGAAGACC |
| HEY1 | TGGATCACCTGAAAATGCTG | CGAAATCCCAAACTCCGATA |
| HES1 | ATAGCTCCCGGCATTCCAAG | GCGCGGTATTTCCCCAACA |
| NRARP | GGGCTGCATAGAAAATTGGA | CCCTTTTTAGCCTCCCAGAG |
| MYC | TGCTCCATGAGGAGACACC | CTTTTCCACAGAAACAACATCG |
| CCND2 | CCTCCAAACTCAAAGAGACCAG | TTCCACTTCAACTTCCCCAG |
| FN14 | CAGACTCTTCCAACCACAAGG | ACCTAGCTTGAGGCTCTCTGTCT |
| VEGF | AGGGCAGAATCATCACGAAGT | AGGGTCTCGATTGGATGGCA |
| HIF1A | CGCAAGTCCTCAAAGCACA | TCAGTGGTGGCAGTGGTAGT |
| BIRC5 | TCTCAAGGACCACCGCATCT | TTTGCATGGGGTCGTCATCT |
| MMP2 | GACCTTGGGAGAAGGCCAAG | CCATCGGCGTTCCCATACT |
| GLI1 | GCCGTGTAAAGCTCCAGTGAACACA | TCCCACTTTGAGAGGCCCATAGCAAG |
| PATCHED | GCTACGACTATGTCTCTCACATCAACT | GGCGACACTTTGATGAACCA |
| GLI3 | TGGTCGAAGAGAGCTGAAGTA | CTGTGGCTGCATAGTGATTGC |
| β-catenin | CGTGGACAATGGCTACTCAAGC | TCTGAGCTCGAGTCATTGCATAC |
| Bmi1 | TGGACTGACAAATGCTGGAGA | GAAGATTGGTGGTGGTTACCGCTG |
| YES1 | GGACAAGGATGTTTCGGCGA | GATCTCGGTGAATATAGTTC |
| NOTCH1 | GGTGAACTGCTCTGAGGAGATC | GGATTGCAGTCGTCCACGTTGA |
| YAP1 | GGTTCGCACATCCTCTCTCC | CAGAAACTCGCCTCAAACGC |
| 18S | AATAGCCTTCGCCATCACTGC | GTG AGG TCG ATG TCT GCT TTCC |
| U6 | CTCGCTTCGGCAGCACA | AACGCTTCACGAATTTGCGT |
| GAPDH | GCCGCATCTTCTTTTGCGTCGC | TCCCGTTCTCAGCCTTGACGGT |

Supplementary Table S1. Primers used in the present study
