# Supplementary material for: circSLC4A7 accelerates stemness and progression of gastric cancer by interacting with HSP90 to activate NOTCH1 signaling pathway
Source: Cell Death Dis. 2023 Jul 20;14(7):452. doi: 10.1038/s41419-023-05976-w (PMC10359325; doi:10.1038/s41419-023-05976-w)
Supplement: Supplementary file 6 — STable 2 [file 41419_2023_5976_MOESM6_ESM.docx]

**Supplementary Table S2：The Types, Dilutions and Sources of Antibodies Used for Western Blotting Analysis**

| **Antibody** | **Working dilution** |  |  | **Species** | **Source -Cat. Number** |
| --- | --- | --- | --- | --- | --- |
| Nanog | 1:1000 |  |  | Mouse monoclonal | Cell Signaling Technology  (Cat. No.4893) |
| OCT4 | 1:1000 |  |  | Rabbit monoclonal | Cell Signaling Technology  (Cat. No. 2750) |
| SOX2 | 1:1000 |  |  | Rabbit monoclonal | Cell Signaling Technology  (Cat. No. 3579) |
| CD44 | 1:1000 |  |  | Rabbit monoclonal | Cell Signaling Technology  (Cat. No. 37259) |
| HSP90 | 1:2000 |  |  | Rabbit polyclonal | Proteintech (Cat. No. 13171-1-AP) |
| HEY1 | 1:2000 |  |  | Rabbit polyclonal | Abcam (Cat. No. ab154077) |
| HES6 | 1:1000 |  |  | Mouse polyclonal | Abcam (Cat. No. ab172800) |
| NOTCH1 | 1:1000 |  |  | Rabbit monoclonal | Cell Signaling Technology  (Cat. No. 3608) |
| β-Actin | 1:1000 |  |  | Rabbit monoclonal | Cell Signaling Technology  (Cat. No. 8457) |
